# GPR176 Is a Biomarker for Predicting Prognosis and Immune Infiltration in Stomach Adenocarcinoma

**DOI:** 10.1155/2023/7123568

**Published:** 2023-04-19

**Authors:** Lin Ni, Shuming Chen, Jianyong Liu, He Li, Hu Zhao, Chunhua Zheng, Yawei Zhang, Hancong Huang, Junjie Huang, Bing Wang, Chengzhi Lin

**Affiliations:** ^1^Department of General Surgery, 900TH Hospital of Joint Logistics Support Force (Fuzong Clinical Medical College) (Former Fuzhou General Hospital), Fuzhou, Fujian, China; ^2^Department of Hepatobiliary Surgery, 900TH Hospital of Joint Logistics Support Force (Fuzong Clinical Medical College) (Former Fuzhou General Hospital), Fuzhou, Fujian, China

## Abstract

Immunotherapy based on immune checkpoint inhibitors (ICIs) is considered to be a promising treatment for stomach adenocarcinoma (STAD), but only a minority of patients benefit from it. It is believed that the poor therapeutic efficacy is attributed to the complex tumor immune microenvironment (TIM) of STAD. Therefore, elucidating the specific regulatory mechanism of TIM in STAD is critical. Previous study suggests that GRP176 may be involved in regulating the pace of circadian behavior, and its role in tumors has not been reported. In this study, we first found that GPR176 was highly expressed in STAD and negatively correlated with patient prognosis. Next, we investigated the relationship between GPR176 and clinical characteristics, and the results showed that the stage is closely related to the level of GPR176. In addition, our further analysis found that GRP176 expression level was significantly correlated with chemotherapeutic drug sensitivity and ICI response. KEGG and GO analyses showed that GPR176 might be involved in stromal remodeling of STAD. Furthermore, we analyzed the association between GPR176 expression and immune implication, and the results revealed that GPR176 was negatively related to the infiltration of various immune cells. Interestingly, GPR176 induced the conversion of TIM while reducing the tumor immune burden (TMB). The expression of GRP176 is closely related to the level of various immunomodulators. Moreover, we performed univariate and multivariate regression analyses on the immunomodulators and finally obtained 4 genes (CRCR4, TNSF18, PDCD1, and TGFB1). Then, we constructed a GRP176-related immunomodulator prognostic model (GRIM) based on the above 4 genes, which was validated to have good predictive power. Finally, we developed a nomogram based on the risk score of GRIM and verified its accuracy. These results suggested that GPR176 is closely related to the prognosis and TIM of STAD. GPR176 may be a new potential target for immunotherapy in STAD.

## 1. Introduction

Stomach adenocarcinoma (STAD) is the most common pathological type of gastric cancer (GC), accounting for more than 95% of all GC [1]. Currently, STAD is the fifth most common malignancy and the third leading cause of cancer-related death globally [2]. To date, the classic treatment strategies for STAD are surgery, chemotherapy, antiangiogenic therapy, and radiation therapy [3]. However, these traditional treatments are increasingly difficult to improve the prognosis of STAD, especially for patients with advanced patients. Therefore, exploring new treatment options is extremely important and urgent to improve the prognosis of STAD.

Immunotherapy based on immune checkpoint inhibitors (ICIs) has steadily developed into a research hotspot for STAD treatment with the advent of a number of therapeutic alternatives [4]. Patients with STAD are now being treated with a range of ICIs, and considerable therapeutic results have been attained [5]. Unfortunately, due to heterogeneity of tumors, only a majority of advanced STAD patients benefit from ICIs [6]. Numerous studies have shown a strong correlation between the efficacy to immunotherapy and the percentage of immune cells in the TIM [7]. Therefore, it is particularly important to elucidate the specific mechanisms regulating TIM in STAD for guiding immunotherapy.

GPR176 is a member of the G protein-coupled receptor family, as a membrane receptor protein, widely involved in the response to hormones, growth factors, and neurotransmitters [8]. Previous study showed that GPR176 is involved in the pace of circadian behavior [8]. However, the function and role of GPR176 in malignant tumors are unknown, especially in TIM.

In this study, we first analyzed the expression of GRP176 in STAD and explored the correlation with patient prognosis and various clinical features. The underlying mechanism of GPR176 regulating STAD progression was preliminarily elucidated. We discovered the effectiveness of GPR176 to predict STAD on chemotherapy drugs and ICIs. We revealed the correlation between GPR176 and immune profile in STAD. Critically, we constructed a GPR176-based prognostic model and demonstrated its accuracy.

## 2. Materials and Methods

### 2.1. Acquisition of STAD Data from the Public Database

TCGA database is an open platform for global users, which contains many types of malignant tumor data. The STAD transcription data was downloaded from TCGA database (https://portal.gdc.cancer.gov/). We applied the corresponding functions in the limma package of R software to process the obtained data. In this study, a total of 371 STAD samples were obtained after excluding samples with incomplete clinical information.

### 2.2. Analysis of Immune Cell Infiltration in STAD

We applied multiple databases to study the relationship between GPR176 and immune cell infiltration, including TIMER, TISIDB, and Cell-Type Identification by Estimating Relative Subsets of RNA Transcripts (CIBERSORT). TISIDB is a web server [9]. CIBERSORT is a novel calculation method that can use the characteristic genes in the transcription group data to mark the type of immune cells. CIBERSORT relied on a matrix file called LM22 to analyze the immune cells in the data to distinguish various immune cells [10].

### 2.3. Prediction of GPR176 Expression on the Effect of ICIs and Chemotherapy Drugs

Immunophenoscore (IPS) is an indicator for predicting reactions to ICIs (anti-CTLA-4 or anti-PD-1) response [11]. This method is quantitatively immune-related genes, including MHC-related molecules, immune checking points, or effector cells and suppressor cells. Finally, the final score is obtained by the average weight. In this study, we analyze the expression level of GPR176 through algorithms and then predict the sensitivity of chemotherapy drugs. The principle of this algorithm is to analyze the different expression genes between the GPR176 high and low expressions. Then, the results were submitted to the CMap (Connectivity Map) database and then analyze the corresponding chemical drugs.

### 2.4. Statistics

In this study, various function packages based on R software are used for calculation and statistical analysis. *p* < 0.05 were considered statistically significant.

## 3. Results

### 3.1. GRP176 Is Highly Expressed and Associated with Poor Prognosis in STAD

**Figure 1:**
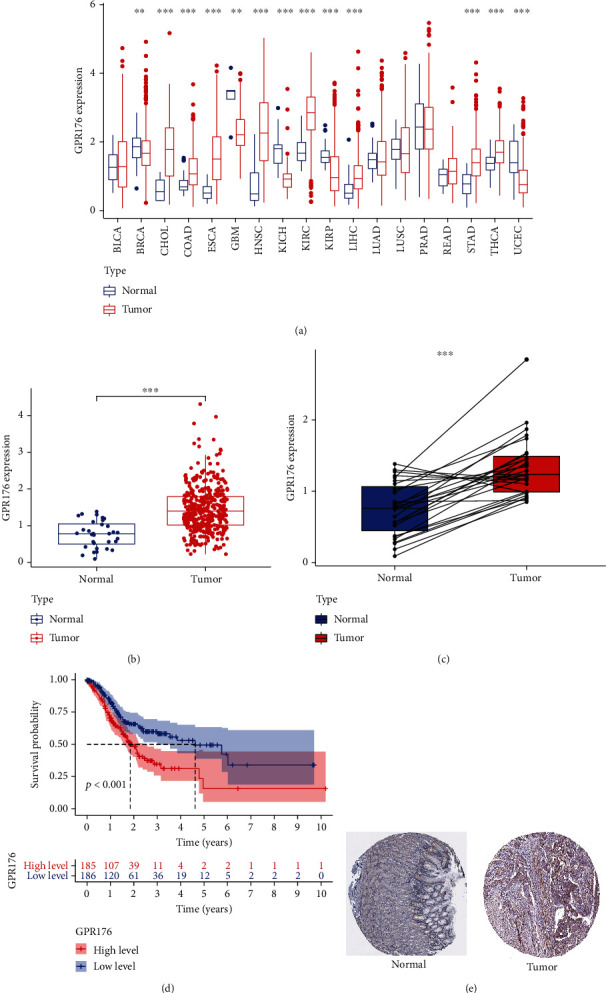
Expression level of GPR176 and its correlation with prognosis in STAD. (a) mRNA expression level of GPR176 in pan-cancer. (b) GRP176 mRNA expression levels in the STAD cohort. (c) Expression level of GRP176 mRNA in STAD and its paired normal tissues. (d) Comparison of overall survival in patients with high and low expressions of GRP176 in STAD. (e) Protein expression levels of GRP176 in STAD and normal gastric tissues.

Given that the role of GRP176 in tumors is unclear, we first evaluated the GPR176 expression levels in pan-cancer tissues. The result showed that the expression of GRP176 was significantly upregulated in most tumors, including STAD (Figures 1(a) and 1(b)). Consistently, further analysis found that GPR176 was significantly higher in STAD than in paired normal gastric tissue (Figure 1(c)). In addition, we performed survival analysis on STAD patients with high and low expressions of GPR176, and the result demonstrated that patients with high expression of GPR176 had a worse prognosis (Figure 1(d)). Moreover, we conducted public database (https://www.proteinatlas.org/) to investigate the protein level of GPR176 in STAD, and the results were consistent with the previous data (Figure 1(e)). The above results indicated that the upregulation of GPR176 in STAD is closely related to patient prognosis.

### 3.2. The Expression Level of GPR176 Is Related to the Clinical Characteristics in STAD

**Figure 2:**
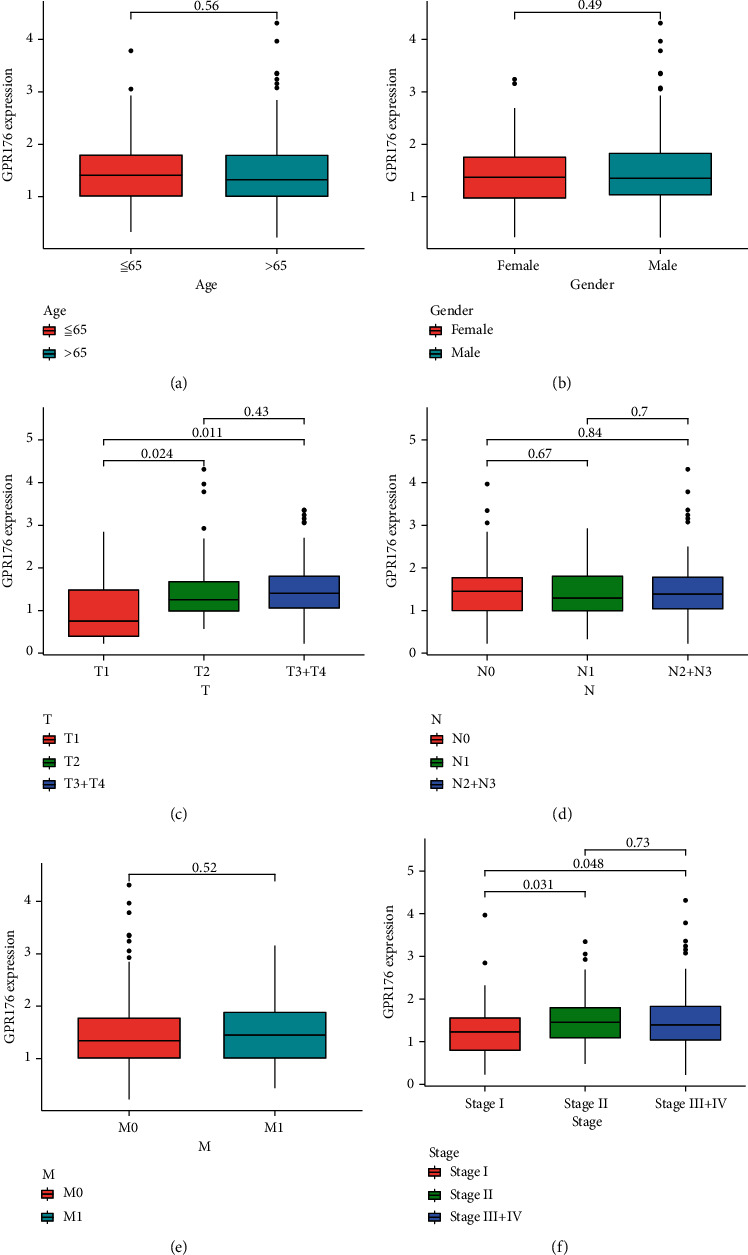
Correlation analysis between GRP176 and STAD clinical features. (a–f) Correlation of clinical features between high and low expressions of GRP176 in STAD.

The previous results demonstrated the close association of GPR176 with poor prognosis of STAD.We intended to analyze the relationship between GPR176 and clinical characteristics. As shown in Figures 2(a) and 2(b), the distribution level of GPR176 does not have significant correlation with gender and age. The level of GPR176 in T2 patients was obviously higher than that in T1 patients, but no further upregulation was observed in T3+T4 (Figure 2(c)). Interestingly, GPR176 levels were not correlated with N and M in STAD patients (Figures 2(d) and 2(e)). In addition, the level of GPR176 in stage II patients was higher than that in stage I patients, but no further upregulation was observed in III+IV (Figure 2(f)).

### 3.3. Prediction of Sensitivity to Immunotherapy and Chemotherapeutics by the Expression Level of GPR176 in STAD

**Figure 3:**
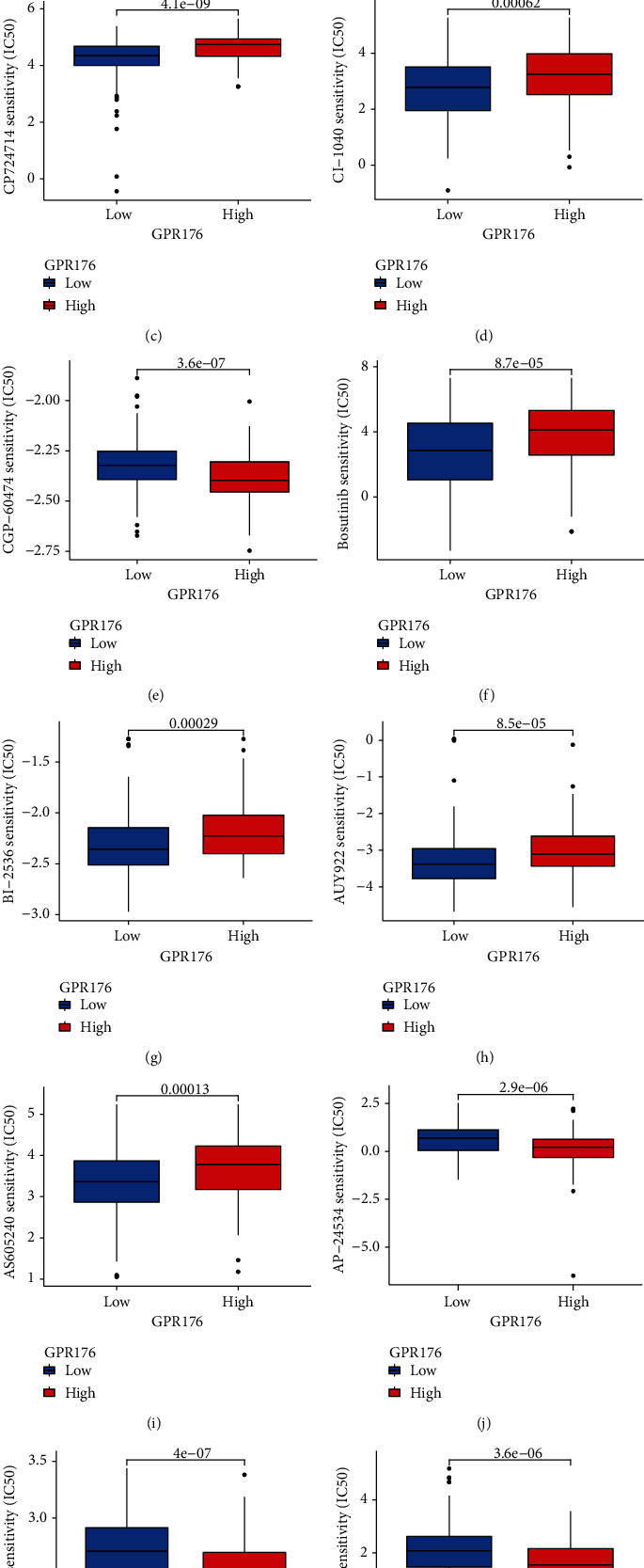
Analysis of GPR176 expression level and drug sensitivity in LIHC. (a–l) Sensitivity comparison of high and low expressions of GRP176 to various chemical drugs.

**Figure 4:**
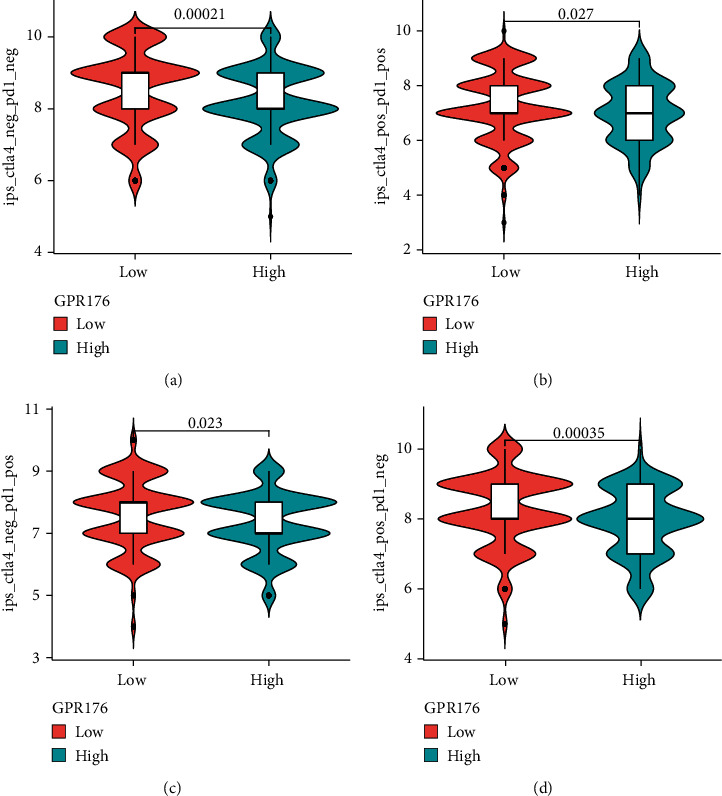
Expression levels of GPR176 and prediction of response to immunotherapy in LIHC. (a) In the case of both CTLA4 and PD-1 double negative, the low GPR176 cohort had higher IPS. (b) In the case of both CTLA4 and PD-1 double positive, the low GPR176 cohort had higher IPS. (c) In the case of CTLA4 negative but PD1 positive, the high GPR176 cohort had higher IPS. (d) In the case of CTLA4 positive but PD1 negative, the high GPR176 cohort had higher IPS.

Our previous analysis implied that upregulation of GPR176 is closely associated with the prognosis of STAD and its pathological features. We further explored whether GPR176 can instruct chemotherapy and immunotherapy in STAD. As shown in Figures 3(a)–3(l), the STAD cohort with high GPR176 expression had lower drug sensitivity to 5-fluoridine, CP724714, CL-1040, bosutinib, BL-2536, AUY922, and AS605240. Conversely, the STAD cohort with low GPR176 expression had lower drug sensitivity to cyclopamine, CGP-60474, AP-24534, and A-770041. In addition, when CTLA4 and PD-1 are negative, the cohort of low GPR176 had a stronger immune response to immunotherapy (Figure 4(a)). When CTLA4 and PD-1 are positive, the cohort of low GPR176 showed a stronger immune response to immunotherapy (Figure 4(b)). In the case of CTLA4 negative but PD1 positive, the high GPR176 cohort showed a stronger immune response to immunotherapy (Figure 4(c)). In the case of CTLA4 positive but PD1 negative, the high GPR176 cohort demonstrated a stronger immune response to immunotherapy (Figure 4(d)).

### 3.4. Enrichment Analysis of GPR176 in STAD

**Figure 5:**
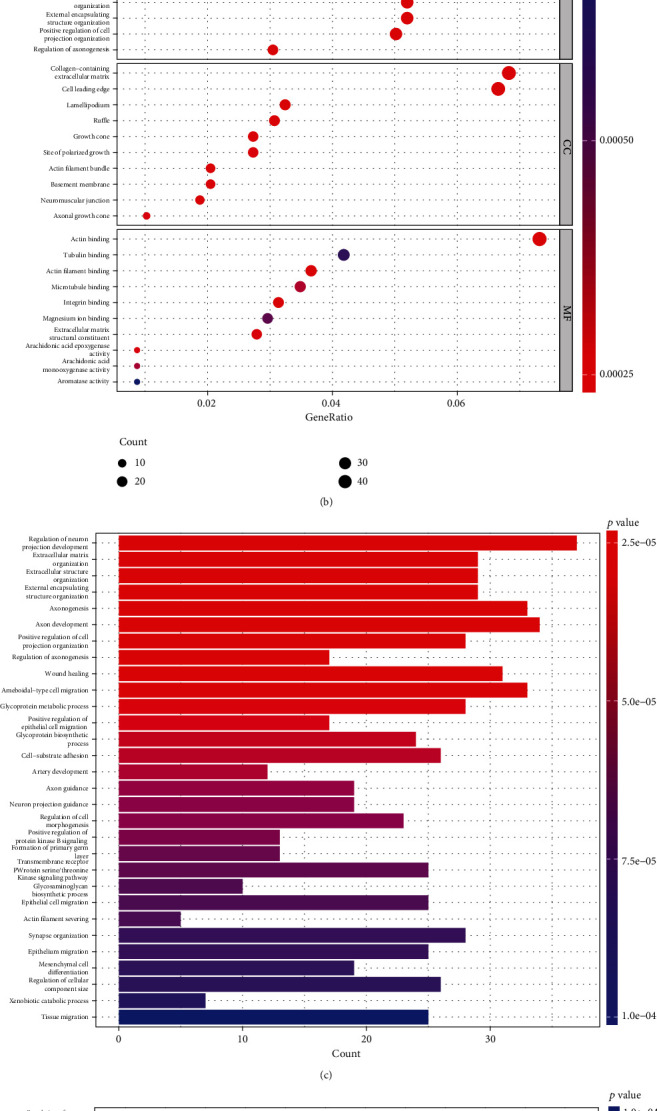
Enrichment analysis of GPR176 expression levels in STAD. (a and b) GO analysis of LIHC cohorts with high and low expressions ofGPR176. (c and d) KEGG analysis of LIHC cohorts with high and low expressions of GPR176.

To initially investigate the potential mechanism of GPR176, we performed KEGG and GO enrichment analyses by differentially expressed genes of GPR176 in STAD. The enrichment analysis of the GO function set showed that GPR176 may participate in cell migration, extracellular matrix ingredients, and cell matrix reshaping (Figures 5(a) and 5(b)). The enrichment analysis of the KEGG function set shows that GPR176 may regulate cell migration, cell matrix, cell adhesion, and migration of epithelial cells (Figures 5(c) and 5(d)).

### 3.5. Correlation Analysis of GPR176 Expression Level and Immune Cell Infiltration

**Figure 6:**
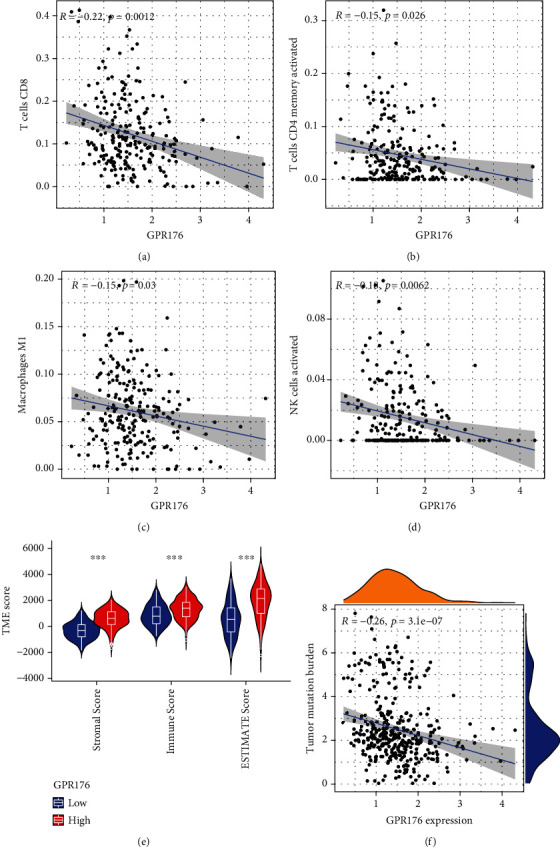
Correlation of GPR176 expression level with immune profile in STAD. (a–d) Correlation between GPR176 expression level and immune cell infiltration in STAD. (e) Correlation between GPR176 expression level and TME score in STAD. (f) Correlation between GPR176 expression level and TMB in STAD.

**Figure 7:**
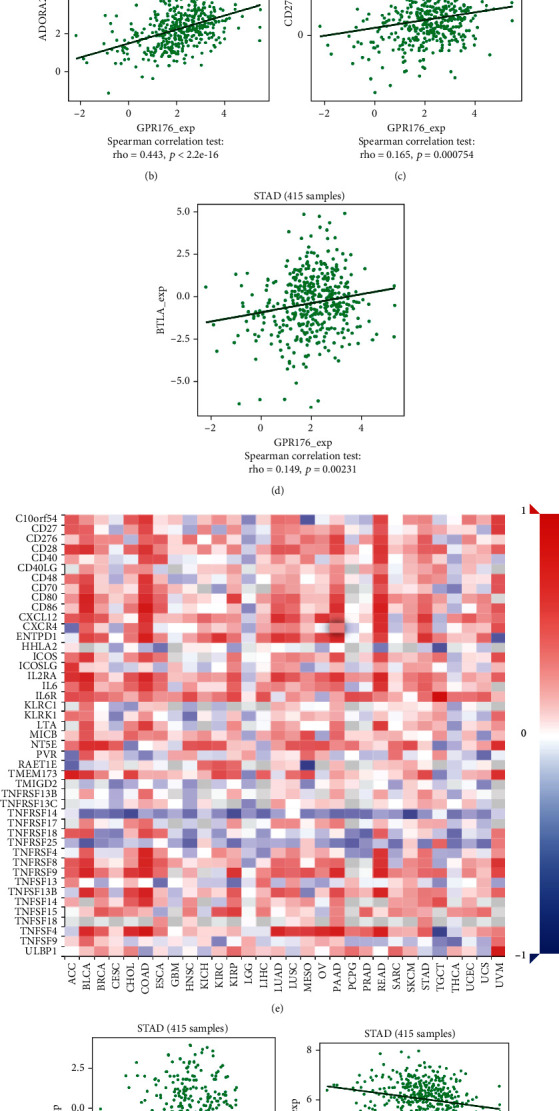
Correlation between GPR17 and immune checkpoints in STAD. (a) Heatmap of correlations between GPR17 expression levels and immunoinhibitor in STAD. (b–d) Correlation between GPR17 expression level and immunoinhibitor. (e) Heatmap of correlations between GPR17 expression levels and immunostimulator in STAD. (f–h) Correlation between GPR17 expression level and immunostimulator.

It is well-known that tumor immunological dysfunction plays a crucial role in tumorigenesis and progression [12]. We analyzed the relationship between GPR176 expression levels and immune profile in STAD. We implemented CIBERSORT to evaluate the relationship between GPR176 and various immune cells. The results indicated that GPR176 is negatively related to the infiltration level of CD8^+^ T cells, CD4 memory activation cells, M1 macrophages, and activated NK cells (Figures 6(a)–6(d)). In addition, we also found that patients with GPR176 have higher TME scores (Figure 6(e)). Interestingly, the expression level of GPR176 was negatively related to the TMB of STAD (Figure 6(f)). These data revealed that GPR176 plays a vital role in regulating the immun cells infiltration of STAD. Furthermore, we further explored whether the GPR176 level alters the expression of each immune checkpoint in STAD. As shown in Figure 7(a), GPR176 was positively related to the expression level of multiple immunoinhibitors, including ADORA2A, CD274, and BTLA (Figures 7(b)–7(d)). Meanwhile, GPR176 was negatively related to the expression level of multiple immunostimulator, including ULBP1, TNFRSF14, and HHLA2 (Figures 7(e)–7(h)). The above results strongly implied that GPR176 plays a vital role in regulating STAD immune profile.

### 3.6. Construction of Risk Score Model Based on GPR176-Related Immunomodulators

**Figure 8:**
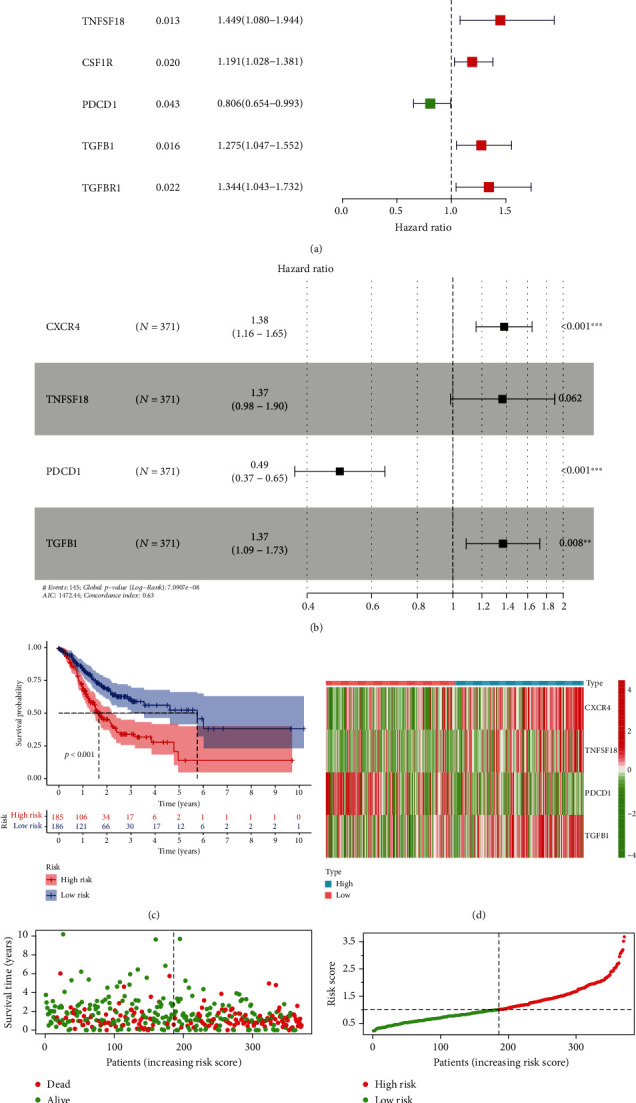
Construction of risk score model derived from GPR176-associated immunomodulators. (a) Univariate regression analysis of GPR176-related immunomodulators on STAD prognosis. (b) Multivariate regression analysis of GPR176-related immunomodulators on STAD prognosis. (c) OS comparison between high- and low-risk cohorts. (d) Distribution plot of immune checkpoints in high- and low-risk cohorts. (e) Distribution plot of survival status of patients according to risk score ranking. (f) Risk score ranking for high- and low-risk patients.

The previous results revealed that the GPR176 participate in regulating the immune function of STAD. We further intended to construct a prognostic model based on GPR176-related immunomodulators. We first conducted univariate and multivariate regression analyses to the obtained GPR176-related immunomodulators, and 4 GPR176-related genes were identified, including CXCR4, TNFSF18, PDCD1, and TGFB1 (Figures 8(a) and 8(b)). Furthermore, we constructed a prognosis model derived from the above 4 GPR176-related immunomodulators (GRIM). We further verified the prediction capabilities of the above model, and the results demonstrated that it has good prediction capabilities (Figures 8(c)–8(f)).

### 3.7. Construction of a Nomogram Based on GRIM

**Figure 9:**
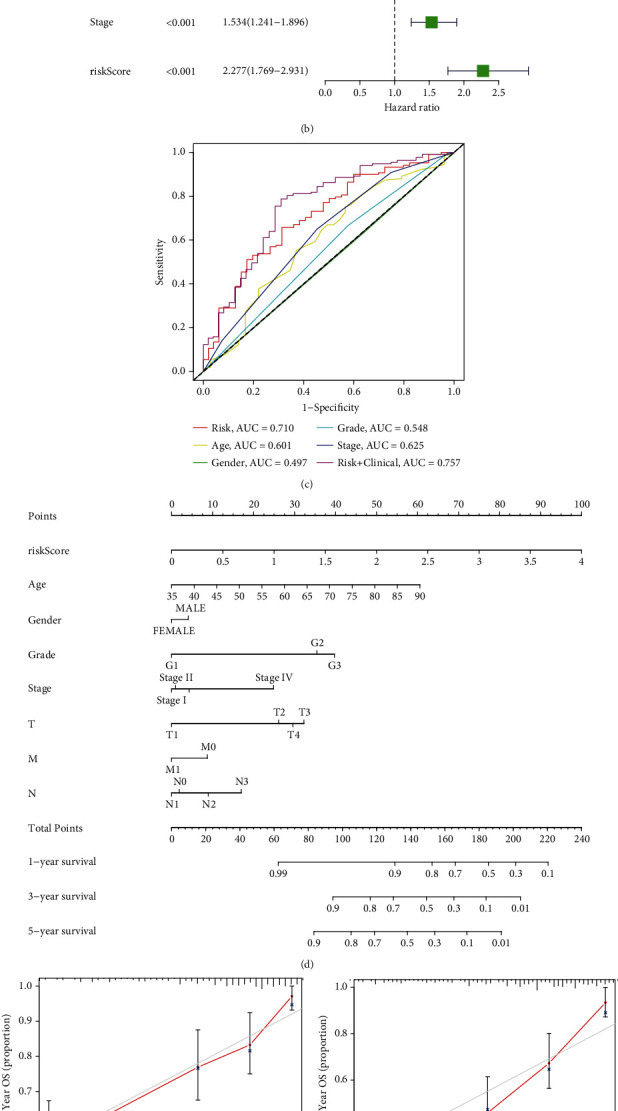
Construction of prognostic nomogram based on the risk scores of GRIM in STAD. (a) Univariate regression analysis of risk scores of GRIM and various clinical characteristics for STAD prognosis. (b) Multivariate regression analysis of risk scores of GRIM and various clinical characteristics for STAD prognosis. (c) ROC curves for risk score (GRIM) and clinical features. (d) Construction of GRIM nomogram and various clinical features in STAD. (e–g) Verification of the accuracy of the nomogram.

We intended to further explore the value of GRIM model in STAD. The risk score of GRIM model is identified as a prognostic risk factor for STAD by univariate and multivariate regression analyses (Figures 9(a) and 9(b)). As shown in Figure 9(c), the area under the ROC curve of the risk score reached 0.710. In addition, we developed a nomogram based on clinical features and risk score of GRIM model to predict the prognosis of STAD. The calibration curve indicated that the nomogram of GRIM model has excellent prognosis prediction capabilities for STAD patients in 1, 2, and 3 years (Figures 9(e)–9(g)).

## 4. Discussion

Immunotherapy brings new hopes for patients with advanced STAD. Therefore, it is particularly important to clarify the specific regulation mechanism of TIM. In this study, we first analyzed the expression of GRP176 in STAD and explored the correlation between its expression level and patient prognosis. We revealed that the level of GPR176 is significantly related to multiple clinical features. In addition, we found that GPR176 can be used to predict the efficacy of STAD on chemotherapy drugs and immunotherapy. KEGG and GO analyses were conducted to initially explore the potential mechanisms by which GPR176 regulates STAD progression. Moreover, we further analyzed the correlation between GPR176 and immune profile of STAD. Finally, we constructed a prognostic model based on GPR176 and verified its accuracy and effectiveness through multiple methods. In the present study, we first revealed that GPR176 was closely related to the prognosis of STAD. GRP176 may participate in the regulation the TIM of patients with STAD.

At present, the TNM classification of malignant tumors is widely used in staging STAD, and its effectiveness and accuracy are confirmed [13]. In this study, we analyzed the correlation between GPR176 and TNM. It was found that the expression level of GPR176 at T2 was significantly upregulated compared to T1, and there was no further increase in T3 and T4. In N and M, there is no difference in expression of GPR176. Interestingly, the expression level of GPR176 at stage II was significantly upregulated compared to stage I. Given the above-mentioned abnormal results, this may be due to the fact that STAD is primarily involved in the regulation of STAD proliferative capacity, or to the insufficient number of samples adopted in this study.

Malignant tumors have extremely complex gene networks, including a series of oncogenes and tumor suppressor genes, which regulate the occurrence and development of tumors [14]. In recent years, prognostic models based on various functional gene sets have emerged to evaluate the prognosis of tumors. In breast cancer, Hong et al. constructed a prognostic model based on the tumor microenvironment-related gene set, and the area under the ROC curve of the model risk score reached 0.67 [15]. Wang et al. analyzed the miRNA data of central lymph node metastasis in papillary thyroid carcinoma and then constructed a differential miRNA prognostic model with an area under the ROC curve of 0.7 [16]. In this study, the area under the ROC curve of the GRP176-based prognostic model we constructed was 0.71. The single-gene model based on GPR176 in this study also had excellent prognostic assessment compared with previous multigene prognostic models. This result indicated that the prognostic model derived from a single gene has a good application prospect.

The present study provided strong evidence to support the important role of GPR176 in STAD, but there are significant shortcomings. All the data in this study were obtained from public databases and lacked in vivo and in vitro validation, we will further confirm this in the follow-up study.

Collectively, our findings revealed a novel role of GRP176 in STAD, and GRP176 may be a promising potential target for STAD immunotherapy.

## Data Availability

The data and result in this study are available from the corresponding authors upon reasonable request.
